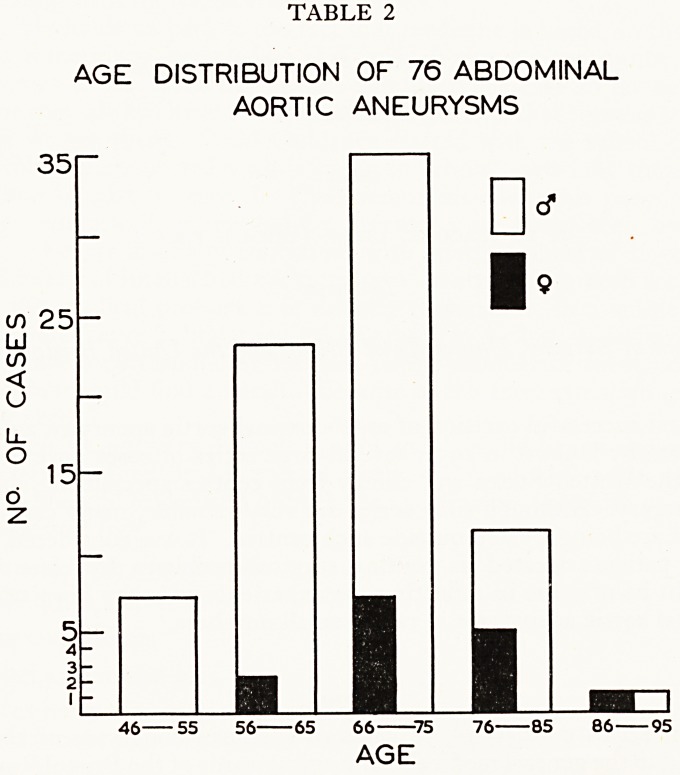# Experience with Abdominal Aortic Aneurysims

**Published:** 1963-04

**Authors:** H. T. John, J. H. Peacock

**Affiliations:** Department of Surgery, University of Bristol and the United Bristol Hospitals; Department of Surgery, University of Bristol and the United Bristol Hospitals


### EXPERIENCE WITH ABDOMINAL AORTIC ANEURYSMS

H. T. JOHN, D.F.C., M.S. (DURH.), F.R.C.S.

*Lecturer*

AND

J. H. PEACOCK, CH.M. (BIRM.), F.R.C.S.

*Lecturer*

(*Department of Surgery, University of Bristol and the United Bristol Hospitals*)

Since the first successful excision of an abdominal aortic aneurysm and reconstruction of the aorta by Dubost in 1952, several large series of cases have been reported, mainly from the United States and chiefly from centres specialising almost entirely in vascular surgery. Although such series are very valuable, many cases of this type have been and are being treated outside such centres. It was considered that a report dealing with patients treated as routine surgical problems by essentially general surgeons might be of value in reflecting the experience of many hospitals which deal with abdominal aortic aneurysms in rather small numbers.

### MATERIAL

The present series consists of 76 cases of fusiform aneurysm of the abdominal aorta, admitted to the general medical and surgical wards of the Bristol Royal Infirmary in a 10 year period between 1952 and 1962. Forty-seven of the aneurysms were uncomplicated and 29 complicated by rupture or threatened rupture at the time of admission (Table 1). In all except eight patients with ruptured aneurysms, the diagnosis

TABLE 1

ARTERIAL ANEURYSMS ADMITTED TO THE BRISTOL ROYAL INFIRMARY 1952 TO 1962

| | |
|---|---|
| Total number arterial aneurysms, all sites | 124 |
| Number of saccular abdominal aneurysms | 76 |
| Uncomplicated | 47 |
| Ruptured | 29 |
| Number of dissecting aneurysms involving the abdominal aorta | 4 |

had been suspected during life and in the majority a firm clinical diagnosis had been made, aided in some cases by aortography or, more often, by straight x-ray or tomography of the abdomen. All the aneurysms were arteriosclerotic in nature, 66 arising below the renal arteries and 9 at or above this level. In one patient, who was not operated upon, the exact site of origin in relation to the renal arteries was not clear. There were 61 men and 15 women in the series and the age range was 47 to 91 years.

The average age of the entire group was 67·8 years, compared with 74·2 years for the women alone. (Table 2).

TABLE 2

CLINICAL PICTURE

*Symptoms.* A detailed history was available in 72 of the 76 cases at the time of admission. In 14 of the 47 uncomplicated cases the aneurysm was symptomless and was discovered either on routine examination or at operation for an unrelated condition. Of the remaining 33 cases, 14 had noticed a pulsatile abdominal swelling, with or without a sensation of throbbing, the remainder had noticed pain of some degree, usually a dull ache, either in the abdomen, back, or both, and radiating to the groin, thigh, or testicle. All the patients with ruptured aneurysms experienced severe pain, either in the back alone (8), in the abdomen (10), or radiating from the abdomen to the back (9). The pain sometimes entered the loins (3), groins (1), or testicle (1) and was occasionally accompanied by vomiting (2) (Table 3).

*Signs.* All the patients with uncomplicated aneurysms had a pulsatile abdominal mass with or without some degree of tenderness over the aneurysm. In the cases complicated by rupture, all showed evidence of haemorrhagic shock amounting in many almost to complete exsanguination and, with the exception of 8 cases in which an erroneous diagnosis was made, a tender pulsatile mass had been found.

TABLE 3

PRESENTING SYMPTOMS IN 72 PATIENTS WITH ABDOMINAL AORTIC ANEURYSM

| *Symptom* | *Number of patients* | | | |
|---|---|---|---|---|
| | | Uncomplicated | | Ruptured |
| Symptomless | | 14 | | |
| Pulsatile swelling | | 14 | | |
| Throbbing sensation | | 5 | | |
| Pain or discomfort | | 29 | | |
| Abdomen | 14 | | | |
| Back | 9 | | | |
| Loins | 1 | | | |
| Groin | 2 | | | |
| Thighs | 2 | | | |
| Testicle | 1 | | | |
| Severe pain | | | | |
| Abdomen | | | | 10 |
| Back | | | | 8 |
| with radiation to: | | | | |
| abdomen | | | 9 | |
| loin | | | 3 | |
| groin | | | 1 | |
| testicle | | | 1 | |
| Vomiting | | | | 2 |

### *Differential Diagnosis*

The clinical diagnosis was initially in error in 8 patients subsequently shown to have a ruptured aneurysm at post-mortem or too late for operative treatment to be considered. The provisional diagnoses made were pancreatitis, biliary colic, perforated viscus and coronary artery occlusion; the error arose in each case because of failure to detect the presence of a pulsatile abdominal mass.

### *Treatment*

Thirty-four of the aneurysms were managed without operation; 19 of these were uncomplicated, 14 were ruptured and there was 1 aneurysm classified as leaking.

In the 19 uncomplicated cases, operation was either refused by the patient or not considered advisable for reasons of age or the presence of severe systemic, pulmonary, or cardiovascular disease. Three of these patients died in hospital, and 7 of the patients are still alive, 1 having survived over five years and the remainder from 6 months to over 4 years. The causes of death in those who died after discharge are shown in Table 4. Fourteen ruptured aneurysms and 1 leaking aneurysm were not operated upon. Five of these patients were moribund on arrival and died before reaching the operating theatre, whilst in eight patients, previously mentioned, the diagnosis was only made immediately before death or at post-mortem. The remaining patient was 91 years of age and was unfit to withstand any major procedure. The patient diagnosed as having a leaking aneurysm involving the renal arteries was 78 years old and his symptoms settled in hospital with sedatives. He was subsequently discharged and is still alive over 1 year afterwards. The other fourteen patients all died from haemorrhage within a few hours to 12 days after admission.

TABLE 4

FOLLOW UP STUDY OF 16 CASES OF UNTREATED UNCOMPLICATED AORTIC ANEURYSM AFTER DISCHARGE FROM HOSPITAL

| *Alive* | | | *Dead* | | | |
|---|---|---|---|---|---|---|
| Patient | Age at diagnosis | Time since diagnosis | Patient | Age at diagnosis | Time between diagnosis and death | Cause of death |
| Sh | 69 | 6 mths. | G | 75 | 1 wk. | Perforated D.U. |
| Sm | 78 | 1 yr., 2 mths. | Wh | 76 | 6 mths. | Carcinoma stomach |
| Du | 70 | 2 yrs. | S | 84 | 7 mths. | Rupture |
| Sh | 79 | 2 yrs., 8 mths. | M | 72 | 1 yr., 1 mth. | Coronary occlusion |
| Ed | 77 | 3 yrs. | Ph | 65 | 1 yr., 3 mths. | Coronary occlusion |
| Da | 62 | 4 yrs., 5 mths. | Mi | 64 | 1 yr., 8 mths. | Rupture |
| Ba | 72 | 5 yrs., 9 mths. | F | 70 | 2 yrs. | Rupture |
| | | | Bo | 57 | 2 yrs., 4 mths. | Rupture |
| | | | H | 59 | 4 yrs., 3 mths. | Coronary occlusion |

### RESULTS OF SURGICAL TREATMENT

Forty-two cases were treated surgically, 28 of these being uncomplicated and 14 complicated by rupture. Five uncomplicated and 2 ruptured aneurysms were treated by the insertion of stainless steel wire. Excision of the aneurysm and replacement by homograft or prosthesis was attempted in 23 cases of intact aneurysms and 12 cases of ruptured aneurysm. The operation was completed in all the intact aneurysms and in 10 of those that had ruptured. Failure to complete the operation in two patients with rupture was due to cardiac arrest occurring immediately after induction of anaesthesia in one patient, and to accidental air embolism from pressure transfusion in another (during an attempt to resuscitate an almost moribund patient).

There were 5 hospital deaths in the 23 uncomplicated cases which were treated by excision and grafting, a mortality rate of 21·7 per cent. The causes of death were renal failure, pulmonary collapse, haemorrhage from the suture line, coronary occlusion and acute tracheo-bronchitis (Table 5). In the 10 ruptured aneurysms in which

TABLE 5

POST-OPERATIVE DEATHS FOLLOWING OPERATION FOR ABDOMINAL AORTIC ANEURYSM

| *Cause of death* | *Uncomplicated Group* | Ruptured *Group* |
|---|---|---|
| Cardiac arrest during anaesthesia | | 3 |
| Cardiac arrest after removal of controlling clamps | | 5 |
| Reactionary haemorrhage | 1 | 1 |
| Coronary occlusion | 1 | |
| Cardiac failure | 1 | 1 |
| Pulmonary collapse | 1 | |
| Renal failure | 1 | |
| Acute tracheo-bronchitis | 1 | |
| Perforated gallbladder | 1 | |
| *Totals* | 7 | 10 |

excision and replacement was possible there were 6 deaths, 5 of which were due to cardiac arrest at the time of, or shortly after, removal of the controlling clamps; and one resulted from reactionary haemorrhage from the aortic suture line. Two ruptured aneurysms were wired, one patient dying of cardiac arrest during the operation and the other 2 days later from cardiac failure. Two of the uncomplicated patients whose aneurysms were wired died, one from undiagnosed peritonitis due to a perforated gallbladder 11 days after operation, the other from a coronary occlusion 2 days after wiring. (Table 6).

TABLE 6

HOSPITAL OUTCOME IN 76 PATIENTS WITH AN ABDOMINAL AORTIC ANEURYSM

| *Site and Type of Aneurysm* | *Group subjected to operation* | | *Group with no operation* | |
|---|---|---|---|---|
| | *Number* | *Deaths* | *Number* | *Deaths* |
| *Below renal arteries* | | | | |
| *Uncomplicated* | | | 14 | 3 |
| Wired | 3 | 1 | | |
| Excised and grafted | 23 | 5 | | |
| *Ruptured* | | | 13 | 13 |
| Wired | 1 | 1 | | |
| Excised, operation not completed | 2 | 2 | | |
| Excised and grafted | 10 | 6 | | |
| *Above renal arteries* | | | | |
| *Uncomplicated* | | | 4 | 0 |
| Wired | 2 | 1 | | |
| *Ruptured* | | | 1 | 1 |
| Wired | 1 | 1 | | |
| *Leaking* | | | 1 | 0 |
| *Unknown relation to renal vessels* | | | | |
| *Uncomplicated* | | | 1 | 0 |

*Post-operative Complications*

Excluding relatively minor complications associated with anaesthesia or intravenous infusion, there were major complications in 7 cases during the immediate post-operative period in patients who subsequently survived and were discharged from hospital. Two patients experienced disruption of the abdominal wound, one developed a transient paraplegia and there were 2 cases of popliteal artery thrombosis, one in a pre-existing aneurysm. In one patient intestinal obstruction occurred 4 days post-operatively from adhesions to a piece of oxycel gauze around the aortic anastomosis. He subsequently suffered perforation of a large gastric ulcer on the 10th day after the original operation and underwent partial gastrectomy, following which he made an uneventful recovery. One patient developed an infected wound which required drainage.

### LONG TERM RESULTS

*Untreated group.* Sixteen patients with uncomplicated aneurysms were discharged from hospital during the period. Seven are still alive; the remainder have died either of rupture of the aneurysm (4), cardiovascular disease (3) or unrelated causes (2) (Table 4).

*Group Treated Surgically.* Twenty-five patients survived operation and were discharged, 22 after excision and grafting and 3 after wiring of the aneurysm. Thirteen are still alive having survived for periods varying from 3 months to 7 years; 12 have died, 2 from rupture of the graft, 1 from ruptured aneurysm of the suture line, one from carcinomatosis, and the remainder from coronary artery occlusion (Table 7).

TABLE 7

LATE RESULTS IN 25 PATIENTS WHO SURVIVED TO LEAVE HOSPITAL AFTER OPERATION ON AN ABDOMINAL AORTIC ANEURYSM

*Alive at time of report*

| | *Patient* | *Age at Operation* | *Time since operation* |
|---|---|---|---|
| *Excised and grafted group* | | | |
| | Ha (ruptured) | 61 | 4 mnths. |
| | Jo | 73 | 1 yr. 2 mnths. |
| | Bo (ruptured) | 60 | 1 yr. 5 mnths. |
| | Gr | 58 | 1 yr. 7 mnths. |
| | En | 71 | 1 yr. 11 mnths. |
| | Hi | 70 | 3 yrs. |
| | Wa | 60 | 3 yrs. 2 mnths. |
| | Gr (ruptured) | 68 | 4 yrs. 2 mnths. |
| | Ba | 72 | 4 yrs. 6 mnths. |
| | Va | 66 | 4 yrs. 6 mnths. |
| | Ph | 75 | 7 yrs. |
| *Wired group* | | | |
| | Gi | 71 | 3 mnths. |
| | Ge | 71 | 3 mnths. |

*Died after discharge*

| *Patient* | *Age at operation* | *Time since operation* | *Cause of death* |
|---|---|---|---|
| *Excised and grafted group* | | | |
| S | 55 | 6 weeks | Ruptured graft |
| Sm | 53 | 3 mnths. | Ruptured graft |
| Da | 58 | 5 mnths. | Aneurysm of suture line |
| T | 59 | 5 mnths. | Coronary occlusion |
| W | 70 | 7 mnths. | Coronary occlusion |
| J | 71 | 7 mnths. | Coronary occlusion |
| R | 57 | 1 yr. 8 mnths. | Coronary occlusion |
| Je | 69 | 2 yrs. | Carcinomatosis |
| B | 65 | 3 yrs. | Coronary occlusion |
| P | 62 | 3 yrs. 6 mnths. | Coronary occlusion |
| Ho (ruptured) | 73 | 6 yrs. 3 mnths. | Coronary occlusion |
| *Wired group* | | | |
| Cr | 70 | 2 yrs. | Coronary occlusion |

### DISCUSSION

The indication for operation in abdominal aortic aneurysm is essentially the threat of rupture of the aneurysm and the consequent danger to life. It is probable that rupture of an aortic aneurysm is inevitable if the patient lives long enough (DeBakey and Crawford 1959), and the classical series of Estes (1950) and Barrett-Boyes (1957) have shown that the majority of untreated patients die within 5 years of diagnosis from the complication. Schatz, Fairbain and Juergens (1961) have disputed the uniformly poor prognosis of untreated aneurysms, and suggest that the presence of heart disease is more often a deciding factor in survival. However, MacVaugh and Brooke (1961), reviewing their own and published series of abdominal aortic aneurysms, indicate that following successful resection and grafting, the expectation of life of patients is essentially that of normal individuals in the same age group. In any event the high mortality rate of ruptured aneurysm in any age group suggests that every effort should be made to treat these aneurysms during the uncomplicated stage of the disease. With standardisation of the surgical technique and post-operative care, it is reasonable to suppose that the operative mortality could be reduced to below 10 per cent. In this context it is noteworthy that 3 of the post-operative deaths that occurred in the uncomplicated group of cases were essentially preventable, and took place in the earlier years of the series, when neither the anaesthetists nor the surgeons were aware of the problems involved in the management of these patients. It is clear, however, that in order to achieve an acceptable mortality rate, a certain degree of selection must be practised, and there remains quite a large group of patients of advanced age, or with severe pulmonary or cardiovascular disease, in whom the risk of a major operative procedure is prohibitive. Several patients of this type have recently presented for treatment with aortic aneurysms which were patently enlarging and on the point of rupture. They have led us to reconsider the question of the introduction of steel wire into the aneurysm in such cases. The objections to this procedure in the past have been associated with the time required to introduce a sufficient quantity of wire into the aneurysm to promote clotting, and the inefficacy of the method when only a small quantity of wire has been inserted. During the past few months however, 3 patients with enlarging abdominal aneurysms, all over 70 years of age, have been treated by wiring of the aneurysm, using a machine capable of introducing 500 to 600 feet of 32-gauge stainless steel wire into the aneurysm within 10 to 15 minutes, the whole operation being completed within an hour.

The early results of this procedure have been gratifying. Obvious thrombosis occurred within the aneurysms; the patients were mobilised in 2 days and all 3 were discharged without complications within 2 weeks. We believe that this method may well have a place in the management of those patients in whom, by reason of age or infirmity, a more major procedure is obviously impracticable and yet treatment of the aneurysm is necessary to save life.

Unfortunately the problem of the ruptured aortic aneurysm remains difficult and the mortality rate is still high. It has been our practice to operate on ruptured aneurysms as soon as possible after admission and to transfuse only enough blood to maintain life and secure a systolic blood pressure of around 80 to 90 mms Hg until control of the aorta has been achieved. The major difficulty we have experienced has not been technical in nature, but has been related to our inability to maintain control of the circulation at the time of removal of the occluding clamps after reconstruction of the aorta. At least 5 of the 6 deaths in this group have occurred at this time. Cardiac arrest occurred despite the exercise of great care in removing the clamps, the use of noradrenaline, and the inflation of pressure cuffs on the thighs before unclamping.

The reason for our inability to control the circulation at this time is not wholly clear, but, as the problem has not been encountered in any of the uncomplicated cases of aneurysm, it appears to be related to the effect of hypotension on such organs as the heart, liver and intestines, as well as to the accumulation of toxic metabolites in the ischaemic muscles of the limbs. In 2 of the 5 deaths, overtransfusion was a contributory factor, though probably not a decisive one. This factor, noted and experienced by others (Mavor, Davidson and Clark 1959, Stallworth, Price, Hughes and Parker 1962), has now largely been eliminated by continuously operative venous pressure monitoring through a polythene catheter inserted in the jugular vein. This recording has proved to be of value as an indication of the level of venous return to the heart during the critical period of removal of the occluding clamps, and it has provided a guide to the optimum rate of transfusion of blood. The rapid infusion of large quantities of cold stored blood may produce a temperature differential leading to ventricular fibrillation. In recent cases this has been avoided by transfusion of blood through a heat exchanger constructed of coils of $\frac{3}{8}$ inch nylon tubing in 30 foot lengths.

*Late results in the surgically treated group*

The late results have been disappointing on the whole because of a high incidence of death fron coronary artery disease. There have been three causes of death other than coronary occlusion; carcinomatosis, rupture of the graft, and rupture of an aneurysm at the aortic suture line. Both ruptured grafts occurred in patients in whom a polyvinyl sponge prosthesis was inserted, and we have not experienced this complication with any other type of replacement. The patient who died of rupture of an aneurysm at the aortic suture line had a stormy convalescence, complicated by intestinal obstruction and a perforated gastric ulcer, both requiring further operative treatment. It is possible that the failure of healing of the suture line was related to the prolonged gastric suction and intravenous therapy necessary in this patient. The fact that the remaining seven patients all died of coronary artery disease, serves to emphasise the generalised nature of the degenerative disease in these patients, and stresses the importance of the presence of cardiovascular disease in assessing the prognosis of patients with abdominal aortic aneurysms.

It is not possible to compare directly the results in the surgically treated patients with those in the group in which operation was withheld, but it is noteworthy that of the original 19 uncomplicated cases which were not operated upon, 7 are still alive, 2 for more than 4 years. Of those who have died, 3 died in hospital of quite unrelated causes, 3 have died of coronary occlusion, and only 4 of rupture of the aneurysm.

SUMMARY

Observations are reported on a series of fusiform abdominal aortic aneurysms, extending over a period of 10 years, between 1952 and 1962. Results in both complicated and uncomplicated cases are analysed and some points in their management and subsequent progress are detailed.

*Acknowledgements*

We should like to thank the physicians and surgeons of the United Bristol Hospitals for allowing us access to the records of patients under their care, and Professor R. Milnes Walker for his assistance with the preparation of this paper.

### REFERENCES

Barrett-Boyes, B. G. (1957). *Lancet*. ***ii.*** 716.
DeBakey, M., Crawford, E. S. (1959). *Mod. Concepts. Cardio-Vasc. Dis.*, **28**, 557.
Dubost, C., Allory, M., Oeconomous, N. (1952). *Arch. Surg.*, **64**, 405.
Estes, J. E. (1950). *Circulation*, **2**, 258.
MacVaugh, H., Brooke, R. (1961). *Surg., Gynae., Obstr.*, **113**, 17.
Mavor, G. E., Davidson, L. D., Clark, C. G. (1959). *Brit. J. Surg.*, **47**, 292.
Schatz, I. J., Fairbairn, J. F., Juergens, J. L. (1961). *Circulation*, **24**, 1032.
Stallworth, J. M., Price, R. G., Hughes, J. C., Parker E. F. (1962). *Ann. Surg.*, **155**, 711.

## Figures and Tables

**Table 2 f1:**